# Analysis of Cardiovascular High-Risk Profile in Middle-Aged Lithuanian Men with Arterial Hypertension from 2009 to 2019

**DOI:** 10.3390/biomedicines13020272

**Published:** 2025-01-23

**Authors:** Vaida Šileikienė, Vilma Dženkevičiūtė, Alma Čypienė, Tautvydas Šlapikas, Roma Puronaitė, Jolita Badarienė, Aleksandras Laucevičius, Eglė Butkevičiūtė, Egidija Rinkūnienė

**Affiliations:** 1 Clinic of Cardiac and Vascular Diseases, Faculty of Medicine, Vilnius University, Ciurlionio str. 21, LT-03101 Vilnius, Lithuania; 2 State Research Institute Centre for Innovation and Medicine, Santariskiu str. 5, LT-03101 Vilnius, Lithuania; 3 Clinic Department of Information Systems, Centre of Informatics and Development, Sauletekio str. 9, LT-10222 Vilnius, Lithuania; 4 Department of Software Engineering, Faculty of Informatics, Kaunas University of Technology, Studentu str. 50, LT-51368 Kaunas, Lithuania

**Keywords:** arterial hypertension, primary prevention, cardiovascular risk factors

## Abstract

**Objective**: The prevalence of arterial hypertension in adult men is 34% worldwide and 52% in Lithuania. This paper aims to provide an overview of the prevalence trends of arterial hypertension and its clinical forms in middle-aged Lithuanian men and to assess the risk profiles of the different groups. **Methods**: This retrospective research study collected data from 52,012 Lithuanian male patients. The study population consisted of men aged 40–55 who participated in the Lithuanian High Cardiovascular Risk Program between 2009 and 2019. **Results**: Of the 52,012 participants, 47.2% (*n* = 24,531) were men with arterial hypertension. The prevalence of arterial hypertension in Lithuanian men decreased between 2009 and 2019 (*p* < 0.001). Before the study visit, 12.6% (*n* = 6583) of men were unaware of their diagnosis, and 8.8% (*n* = 4555) of diagnosed cases were untreated. In addition, 2.6% (*n* = 1334) of the men had resistant arterial hypertension. Significant differences in most general characteristics were found between the groups with arterial hypertension. Waist circumference increased from 92.8 ± 10.7 cm in men without arterial hypertension to 109 ± 13.3 cm and in men with resistant arterial hypertension (*p* < 0.001), and mean triglyceride levels increased from 1.55 ± 1.22 mmol/L to 2.32 ± 1.82 mmol/L in these groups (*p* < 0.001). Obesity (63.6%), unhealthy diet (74.7%), physical inactivity (62.9%), and diabetes (25.2%) were more common in the group with resistant arterial hypertension than in all other groups (*p* < 0.001). Meanwhile, dyslipidaemia was the most common risk factor in all groups (from 83.2% in men without arterial hypertension to 92.6% in men with resistant hypertension). **Conclusions**: Almost half of middle-aged men had arterial hypertension, with prevalence decreasing from 2009 to 2019. Significant changes in cardiometabolic characteristics were observed in newly diagnosed cases of arterial hypertension. These changes are even more notable in men with resistant hypertension compared to a non-resistant form. Most cardiovascular risk factors were most prevalent in over 50% of resistant hypertension cases, with dyslipidaemia being the most common risk factor in the entire male sample.

## 1. Introduction

Cardiovascular disease is the most common cause of death worldwide, causing around 17.9 million deaths each year. According to the World Health Organization, four out of five cardiovascular-related deaths are due to heart attacks and strokes, with one-third of these occurring in people under the age of 70 [1]. In Lithuania, almost 50% of all deaths are attributed to cardiovascular events [2]. These mortality rates could be significantly reduced by early detection and effective treatment of cardiovascular risk factors such as dyslipidaemia, elevated blood glucose levels, and smoking. This is particularly important for the most prevalent modifiable cardiovascular risk factor, arterial hypertension, which is often referred to as the “silent killer”, affecting more than 1.3 billion adults worldwide [3].

High blood pressure is closely associated with an increased risk of stroke, heart failure, coronary heart disease, ischemic heart disease, and kidney failure. The risk is exceptionally high in people who develop arterial hypertension before the age of 45 [4]. Although arterial hypertension is rarely an isolated disease, it is often associated with other risk factors, including genetic, gender, age, and environmental factors. It is also closely related to several cardiovascular risk factors such as metabolic syndrome, diabetes, smoking, poor dietary habits, obesity, alcohol and tobacco consumption, and physical inactivity [5].

Globally, the prevalence of arterial hypertension in adult men is 34%, while in Lithuania it reaches 52% [3,6]. Among men with hypertension, only 49% are diagnosed, 38% receive treatment, and only 18% have their blood pressure successfully controlled [3]. Alarmingly, 51% of men with arterial hypertension are unaware of their condition, a figure that is 10% higher than in the female population [3].

It is estimated that increasing the rate of successfully treated hypertension to 50% between 2023 and 2050 could prevent 76 million deaths worldwide [6]. However, progress towards achieving the stated goal is hampered by numerous factors. The high prevalence of other cardiovascular risk factors in men with arterial hypertension and the lack of awareness of their diagnosis are just a few examples. Addressing these challenges through comprehensive treatment strategies is critical to improving men’s cardiovascular health and the whole population’s health.

The aim of this study is to provide an overview of the prevalence trends of arterial hypertension and its clinical forms in middle-aged Lithuanian men and to evaluate the cardiovascular risk profiles of different groups.

## 2. Materials and Methods

### 2.1. Study Design and Population

This retrospective study analysed the data of 52,012 Lithuanian male patients aged 40–55 who participated in the Lithuanian High Cardiovascular Risk Primary Prevention Programme (LitHiR) between 2009 and 2019. The programme, active since 2006, involves approximately 91.6% of all primary healthcare centres in Lithuania.

Patients were screened for cardiovascular risk factors, including lifestyle habits (physical activity, diet, smoking), cardiovascular history (personal and family) and anthropometric measurements (height, weight, waist circumference, body mass index (BMI), blood pressure, and heart rate). Additional investigations included blood tests to measure glucose, total cholesterol (TC), high-density lipoprotein (HDL) and low-density lipoprotein (LDL) cholesterol, and triglycerides to determine the prevalence of metabolic syndrome in high-risk patients.

Blood pressure was measured on the dominant arm at heart level after the patient had been sitting for at least five minutes. Physicians were advised to obtain three measurements and use the average.

Arterial hypertension was diagnosed if systolic blood pressure was ≥140 mmHg and diastolic blood pressure was ≥90 mmHg or if hypertension had been previously documented. Newly diagnosed arterial hypertension referred to cases identified during screening that did not have a documented diagnosis before participation in the primary prevention programme. Previously diagnosed but untreated arterial hypertension referred to patients with documented hypertension but without prescribed treatment. Resistant arterial hypertension was defined as uncontrolled blood pressure (>140/90 mmHg) despite the use of three different classes of antihypertensive medication, including diuretics. Non-resistant arterial hypertension referred to medically treated instances that do not meet the criteria for resistant hypertension.

Abdominal obesity was assessed by waist circumference. The correct place to measure your waist is halfway between your lowest rib and the top of your hipbone. This is roughly in line with your belly button. Abdominal obesity was defined as a waist circumference greater than 102 cm in men.

Dyslipidaemia was diagnosed if one or more blood lipid values were outside the normal range: total cholesterol > 5 mmol/L, LDL cholesterol > 3 mmol/L, HDL cholesterol < 1 mmol/L, or triglycerides > 1.7 mmol/L.

Based on the data collected, patients were divided into five groups according to their arterial hypertension status, and the prevalence of cardiovascular risk factors within each group was analysed:

– Men without arterial hypertension;
– Men with undiagnosed arterial hypertension;
– Men with diagnosed but untreated arterial hypertension;
– Men with non-resistant arterial hypertension;
– Men with resistant arterial hypertension.

### 2.2. Statistical Methods

Continuous variables were expressed as mean ± standard deviation (±SD). ANOVA or the non-parametric Kruskal–Wallis test was used to compare these variables between the different forms of hypertension, followed by pairwise comparisons using either the *t*-test or the non-parametric Mann–Whitney *U* test as appropriate. Categorical variables were presented as absolute frequencies (*n*) and percentages (%), with differences between groups assessed using the χ^2^ test or Fisher’s exact test. Trends in the prevalence of different clinical forms of arterial hypertension from 2009 to 2019 were analysed using the Cochran–Armitage test for trends (also known as the χ^2^ test for trends). A significance level of *p* < 0.05 was set for all statistical tests. The analysis was performed with the statistical software “R” (version 4.4.1), while the tables and graphs for visualisation and presentation of the data were created with “Microsoft Word for Microsoft 365”.

## 3. Results

### 3.1. Prevalence of the Different Clinical Forms of Arterial Hypertension in Men from 2009 to 2019

**Figure 1:**
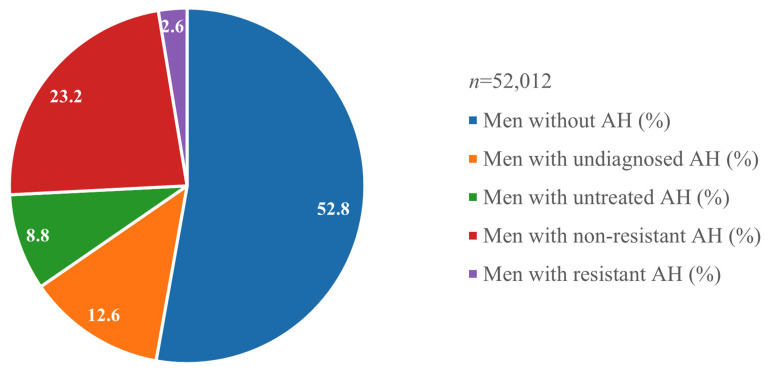
Prevalence of the different clinical forms of arterial hypertension in men.

A total of 52,012 men were examined between 2009 and 2019 as part of the prevention program. Of these, 52.8% (*n* = 27,481) had no diagnosis of arterial hypertension. In 12.6% (*n* = 6583) of cases, arterial hypertension was detected during screening, while 8.8% (*n* = 4555) had been previously diagnosed but did not receive treatment. In addition, 23.2% (*n* = 12,059) of cases were diagnosed and responded to treatment, with 2.6% (*n* = 1334) of participants having resistant arterial hypertension (Figure 1). The overall prevalence of arterial hypertension in male participants was 47.2%, and 74.6% of these patients, excluding newly diagnosed cases, were on treatment. Of the treated cases, 9.96% developed resistant arterial hypertension.

**Figure 2:**
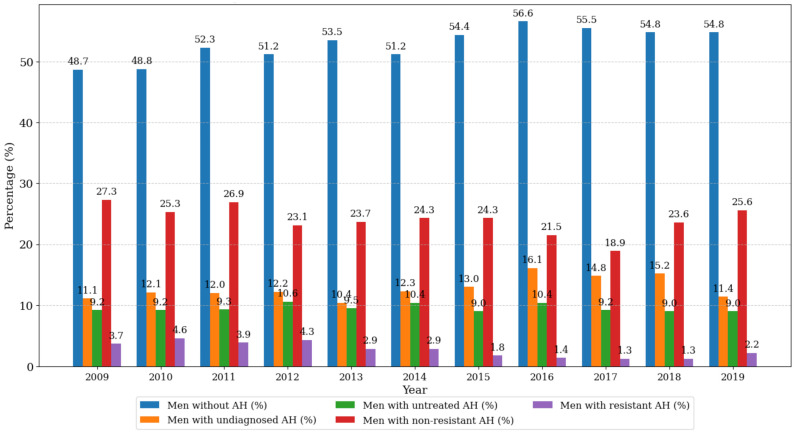
Prevalence of the different clinical forms of arterial hypertension in men in 2009–2019, *p* < 0.001.

There was a stable and significant increase in the proportion of men without arterial hypertension, rising from 48.7% in 2009 to 54.8% in 2019 (*p* < 0.001). From 2018, there was also a notable decrease in untreated cases of diagnosed arterial hypertension and an increase in non-resistant forms (*p* < 0.001) (Figure 2, Table S1).

### 3.2. General Characteristics in Men

The lowest mean age was observed in men without arterial hypertension, at 46.2 ± 4.43 years, while the highest mean age was among men with resistant arterial hypertension, at 48.4 ± 4.19. Men without arterial hypertension had the lowest mean arterial pressure, of 123/77.7 ± 7.24/5.46 mmHg, whereas men with resistant arterial hypertension had the highest mean arterial pressure, at 151/92.4 ± 17.1/10.4 mmHg.

Significant changes in general characteristics were observed between different arterial hypertension groups. The lowest average BMI was recorded in men without arterial hypertension, at 26.4 ± 3.87 kg/m^2^, with BMI increasing progressively across different arterial hypertension groups (*p* < 0.001). A notable increase in BMI was observed between men with non-resistant arterial hypertension, at 30.3 ± 5.07 kg/m^2^, and those with resistant arterial hypertension, at 32.8 ± 5.57 kg/m^2^, respectively (*p* < 0.001). Similar trends were seen in waist measurements, where the mean waist size increased from 92.8 ± 10.7 cm in men without arterial hypertension to 97.1 ± 11.4 cm in men with newly diagnosed arterial hypertension (*p* < 0.001). Comparing men with treatment-responsive and resistant arterial hypertension, waist measurements increased from 103.7 ± 12.9 cm to 109 ± 13.3 cm (*p* < 0.001).

**Figure 3:**
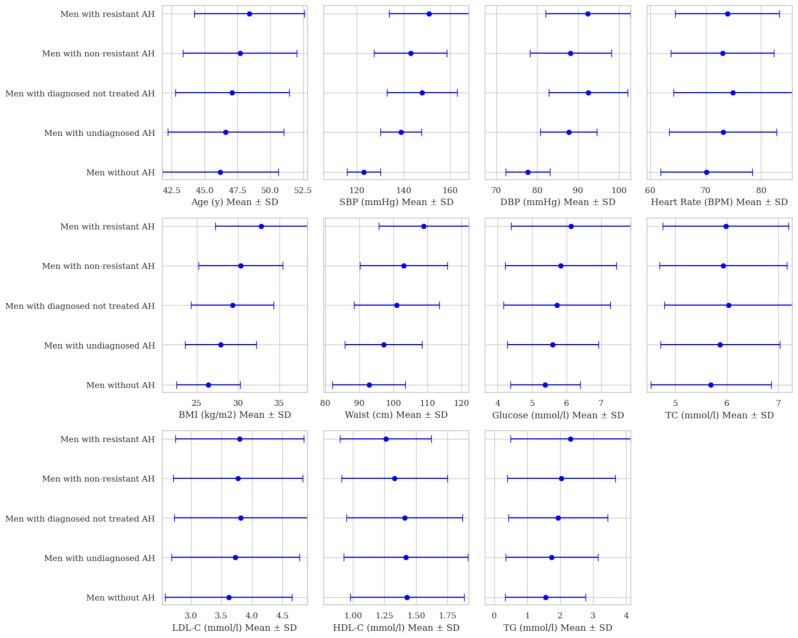
General characteristics in men with and without arterial hypertension. Abbreviations: SBP—systolic blood pressure; DBP—diastolic blood pressure, BPM—beats per minute, BMI—body mass index, TC—total cholesterol, LDL-C—low-density lipoprotein cholesterol, HDL-C—high-density lipoprotein cholesterol, TG—triglycerides.

Mean glucose levels also significantly increased from 5.38 ± 1.01 mmol/L in men without arterial hypertension to 6.12 ± 1.74 mmol/L in men with resistant arterial hypertension (*p* < 0.001). Mean triglyceride levels increase from 1.55 ± 1.22 mmol/L in men without arterial hypertension to 2.32 ± 1.82 mmol/L in those with resistant arterial hypertension (*p* < 0.001). Differences in mean LDL cholesterol levels in most groups with arterial hypertension were insignificant (*p* > 0.05). Meanwhile, changes in HDL cholesterol were insignificant comparing only groups with newly diagnosed and untreated arterial hypertension cases (*p* = 0.464) (Figure 3, Table S2).

### 3.3. Cardiovascular Risk Factors in Men with and Without Arterial Hypertension

The highest prevalence of almost all cardiovascular risk factors was observed in men with resistant arterial hypertension, including decreased HDL cholesterol (47.1%), increased triglyceride levels (56.8%), insufficient physical activity (63.6%), unhealthy dietary habits (74.9%), and a high prevalence of obesity (66.8%) and diabetes (25.9%). The most remarkable differences in the prevalence of risk factors were found between men without arterial hypertension and those with resistant arterial hypertension. For example, 15.9% of men without arterial hypertension were obese compared with 66.8% of men with resistant arterial hypertension (*p* < 0.001). Elevated triglyceride levels were found in 28.5% of men without arterial hypertension, while 56.8% of men with resistant arterial hypertension had hypertriglyceridaemia (*p* < 0.001).

Dyslipidaemia was the most common risk factor in all participants. It affected 87.7% of men without arterial hypertension; and this increased to 95.5% in men with resistant arterial hypertension. The prevalence of diabetes was 6.29% in men without arterial hypertension but increased significantly in men with arterial hypertension: it was 16.0% in men with untreated arterial hypertension and 25.9% in men with resistant arterial hypertension (*p* < 0.001).

**Figure 4:**
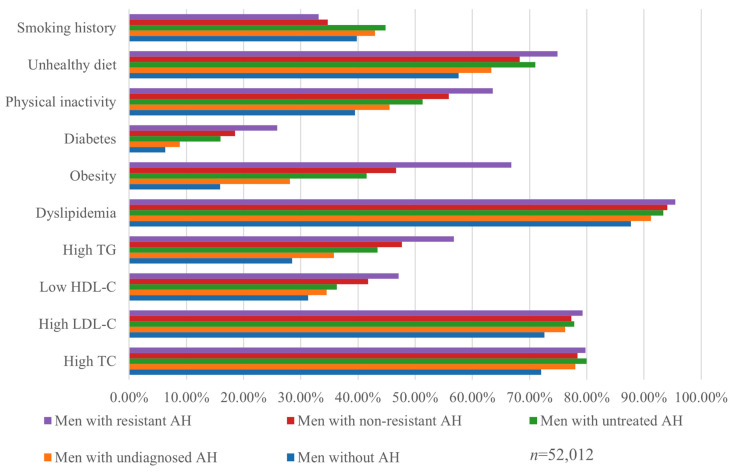
Cardiovascular risk factors in men with and without arterial hypertension. Abbreviations: TG—triglycerides, HDL-C—high-density lipoprotein cholesterol, LDL-C—low-density lipoprotein cholesterol, TC—total cholesterol.

Lifestyle risk factors were also common in the study groups. In the men without arterial hypertension, the prevalence of poor dietary habits was 57.6%, smoking was 39.8%, and lack of physical activity was 39.5%. The prevalence of most risk factors increased significantly when comparing men without arterial hypertension to those with various forms of arterial hypertension (*p <* 0.001). The highest rates of smoking were observed in men with newly diagnosed arterial hypertension and untreated cases of arterial hypertension (43.0% and 44.8%, respectively, *p* = 0.064) (Figure 4, Table S3).

## 4. Discussion

Worldwide, the prevalence of arterial hypertension in 2019 was 32% for women and 34% for men aged 30–79 years [3]. Canada reported some of the lowest rates, with 20% of women and 24% of men affected. In contrast, Central and Eastern European countries such as Poland (55%), Lithuania (54%), Latvia (49%), and the Czech Republic (49%) were among the nations with the highest prevalence of hypertension in men, well above the global average of 34% [3].

In Canada, the prevalence of arterial hypertension in men was 23.6% in 2013, an increase compared to previous years, although awareness and treatment outcomes have improved over the same period [7]. The low prevalence may be surprising, as it seems to be more challenging to access care in Canada than in Lithuania. In 2016, 93% of Canadians reported access to care, while only 1.5% of Lithuanians faced challenges while seeking care in 2019 [8,9]. Between 2007 and 2015, 39.2% of Canadian men reported not getting enough exercise, 77.5% had poor dietary habits, 69.5% were obese, and 26.9% were smokers [10]. In 2018–2019, 56.7% of men over the age of 40 were diagnosed with dyslipidaemia [11]. While smoking rates and physical inactivity declined by 2018, rates of obesity, diabetes, and hypertension have continued to rise since 2001 [12]. These findings may suggest a notable association between dyslipidaemia and arterial hypertension. While self-reported lifestyle habits among Canadian men are broadly similar to those of Lithuanian men, the lower prevalence of dyslipidaemia in Canada should be considered as one of several factors contributing to lower prevalence of arterial hypertension in the Canadian population.

Despite cultural and health differences, Central Eastern European countries, including the Baltics, face similar challenges in terms of cardiovascular risk factors. The LIPIDOGRAM2015 study in Poland, which analysed data from 5034 male patients in primary care, found that 65% of men had hypertension, 88% had dyslipidaemia, and 13% had diabetes. Abdominal obesity was prevalent in 73% of men, and 59% had a history of smoking [13]. A study from the Czech Republic found that 43% of middle-aged men suffered from arterial hypertension, with 8% unaware of their diagnosis. Diabetes was diagnosed in 11% of both men and women. Dyslipidaemia was the most common risk factor, affecting 39% of men. The study also found a significant association between arterial hypertension and other cardiovascular risk factors, including diabetes and dyslipidaemia [14]. In Latvia, 2016 men were interviewed about their cardiovascular risk factors, and 976 men underwent physical examinations as part of a two-part cross-sectional study conducted in 2019–2020. Self-reported rates of hypertension and high cholesterol were 15.9% and 15.4%, respectively, while 40.4% of participants were current smokers [15]. However, objective testing revealed that 40.3% of men had arterial hypertension, 63.1% had LDL cholesterol levels of 3 mmol/L or more, and 60.7% had elevated triglyceride levels [15]. These findings highlight the worrying underestimation of personal health risks that could significantly impact the prevalence and management of cardiovascular disease in men.

This study found that 46.8% of middle-aged men in Lithuania suffered from arterial hypertension. Remarkably, 26.7% of these men were newly diagnosed with arterial hypertension during screening, suggesting that a significant proportion of participants were previously unaware of their cardiovascular health status. Of those with arterial hypertension, 7 out of 10 were receiving treatment, while a quarter of previously diagnosed men were not receiving treatment for AH. Of all treated cases, 11.7% were classified as treatment-resistant. Despite the high overall prevalence, the study observed a decline in arterial hypertension over the decade, with a marked increase in treated cases and a decrease in untreated cases, particularly from 2018 onwards.

Dyslipidaemia was the most prevalent cardiovascular risk factor in all groups of middle-aged men in Lithuania, alongside elevated cholesterol levels and self-reported poor dietary habits. The highest prevalence of elevated triglycerides, insufficient physical activity, and other risk factors was found in men with resistant arterial hypertension, except for smoking. Smoking was more common in men without arterial hypertension or those with untreated hypertension. The most significant differences between men without arterial hypertension and men with resistant arterial hypertension were observed in the prevalence of obesity, elevated triglyceride levels, and the presence of diabetes.

The cardiovascular risk factors are typically classified as modifiable or non-modifiable. This study focuses on the prevalence of modifiable factors, including dyslipidaemia, diabetes, obesity, and lifestyle habits such as physical inactivity, poor dietary habits, and smoking. Arterial hypertension itself is considered a modifiable risk factor for other cardiovascular diseases. However, the aforementioned risk factors play a crucial role in the pathology and aetiology of arterial hypertension. A linear relationship between sodium intake and high blood pressure has been established in multiple studies, with salt reduction proving effective in lowering blood pressure among salt-sensitive individuals [16,17,18]. Physical activity is another well-documented determinant of arterial hypertension, with moderate levels of physical activity shown to reduce the risk of hypertension by 6% [19]. Furthermore, a healthy diet and regular physical activity are vital for preventing diabetes and dyslipidaemia, which are considered as significant contributors to the development of arterial hypertension [20,21,22]. Therefore, the mentioned factors should always be addressed both as preventative and management measures for cardiovascular diseases and cardiometabolic disorders such as diabetes, dyslipidaemia, and arterial hypertension.

### Limitations

This study focuses exclusively on men aged 40–55 years, as the LitHiR primary prevention programme includes men at this age because they are at the highest risk of developing cardiovascular disease. The inclusion criteria for cases of resistant arterial hypertension did not take into account pseudo-resistant or secondary arterial hypertension; thus, caution is advised when interpreting these results. In addition, this study did not consider psychosocial, socioeconomic, and other risk factors nor was there an objective assessment of physical activity, diet, and smoking habits, as these were based solely on self-reported data. As previously mentioned in the discussion, self-reported data should be interpreted cautiously due to potential biases, including underestimating personal health issues and harmful lifestyle habits. Moreover, without objective measurements of these behaviours (e.g., using physical activity trackers or dietary assessments), it is difficult to verify the accuracy of the self-reported data, which limits the accuracy of the study’s conclusions. Therefore, caution should be exercised when interpreting the association between self-reported lifestyle factors and cardiovascular health in this sample. Finally, as the study focuses exclusively on middle-aged men, the results may not be generalizable to other demographic groups, including women or younger people. They may not fully represent the wider Lithuanian population.

## 5. Conclusions

The study highlights the prevalence of arterial hypertension and its trends in middle-aged Lithuanians, as well as the profile of concomitant cardiovascular risk factors in different case groups. According to the results, almost half of middle-aged men (49.8%) suffered from arterial hypertension, with the prevalence decreasing by 6% in the period 2009–2019. However, more than 10% of middle-aged Lithuanian men were unaware of their AH diagnosis, and a quarter of diagnosed cases were untreated. In addition, worrying changes in cardiometabolic characteristics were observed in the newly diagnosed cases of arterial hypertension. These changes were even more pronounced in men with resistant hypertension compared to a non-resistant form. Significant metabolic changes, such as changes in waist circumference, BMI, glucose, and lipid levels, were observed in men with and without AH. Most cardiovascular risk factors, such as unhealthy diet, physical inactivity, obesity, dyslipidaemia, and high low-density lipoprotein cholesterol and total cholesterol levels, were most prevalent in over 50% of resistant hypertension cases, with dyslipidaemia being the most common risk factor in the entire male sample.

## Data Availability

The datasets used and analysed during the current study are available from the corresponding author upon reasonable request.

## Supplementary Materials

The following supporting information can be downloaded at: https://www.mdpi.com/article/10.3390/biomedicines13020272/s1, Table S1: Prevalence of the different clinical forms of arterial hypertension in men in 2009–2019; Table S2: General characteristics in men with and without arterial hypertension; Table S3: Cardiovascular risk factors in men with and without arterial hypertension.

## Abbreviations

The following abbreviations are used in this manuscript:

SBP	Systolic blood pressure
DBP	Diastolic blood pressure
BPM	Beats per minute
BMI	Body mass index
TC	Total cholesterol
LDL-C	Low-density lipoprotein cholesterol
HDL-C	High-density lipoprotein cholesterol
TG	Triglycerides
AH	Arterial hypertension
