# Supplementary material for: Analysis of Cardiovascular High-Risk Profile in Middle-Aged Lithuanian Men with Arterial Hypertension from 2009 to 2019
Source: Biomedicines. 2025 Jan 23;13(2):272. doi: 10.3390/biomedicines13020272 (PMC11852000; doi:10.3390/biomedicines13020272)
Supplement: Supplementary file 1 [file biomedicines-13-00272-s001.zip › biomedicines-3425256-supplementary.pdf]

Analysis of Cardiovascular High-Risk Profile in Middle-Aged Lithuanian Men with Arterial Hypertension from 2009 to 2019

Vaida Šileikienė [1], Vilma Dženkevičiūtė [1], Alma Čypienė [1,2], Tautvydas Šlapikas [1,*], Roma Puronaitė [3], Jolita Badarienė [1], Aleksandras Laucevičius [1], Eglė Butkevičiūtė [4] and Egidija Rinkūnienė [1]

[1] Clinic of Cardiac and Vascular Diseases, Faculty of Medicine, Vilnius University, Ciurlionio str. 21, LT-03101 Vilnius, Lithuania
[2] State Research Institute Centre for Innovation and Medicine, Santariskiu str. 5, LT-03101 Vilnius, Lithuania
[3] Clinic Department of Information Systems, Centre of Informatics and Development, Sauletekio str. 9, LT-10222 Vilnius, Lithuania
[4] Department of Software Engineering, Faculty of Informatics, Kaunas University of Technology, Studentu str. 50, LT-51368 Kaunas, Lithuania
* Correspondence: tautvydas.slapikas@gmail.com

Supplementary Materials

Table S1. Prevalence of the different clinical forms of arterial hypertension (AH) in men in 2009-2019.

	**2009 (*n*=4,251)**	**2010 (*n*=4,494)**	**2011 (*n*=5,383)**	**2012 (*n*=4,575)**	**2013 (*n*=5,071)**	**2014 (*n*=4,970)**	**2015 (*n*=5,186)**	**2016 (*n*=4,233)**	**2017 (*n*=4,217)**	**2018 (*n*=3,886)**	**2019 (*n*=5,746)**	***p* overall**
	n (%)	*n* (%)	*n* (%)	*n* (%)	*n* (%)	*n* (%)	*n* (%)	*n* (%)	*n* (%)	*n* (%)	*n* (%)	*p*-value
**Men without AH**	2,096 (48.7%)	2,191 (48.8%)	2,817 (52.3%)	2,342 (51.2%)	2,715 (53.5%)	2,544 (51.2%)	2,811 (54.2%)	2,303 (54.4%)	2,385 (56.6%)	2,156 (55.5%)	3,148 (54.8%)	<0.001
**Men with undiagnosed AH**	472 (11.1%)	544 (12.1%)	646 (12.0%)	559 (12.2%)	528 (10.4%)	611 (12.3%)	672 (13.0%)	680 (16.1%)	623 (14.8%)	592 (15.2%)	656 (11.4%)	
**Men with diagnosed not treated AH**	390 (9.2%)	413 (9.2%)	500 (9.3%)	487 (10.6%)	481 (9.5%)	516 (10.4%)	468 (9.0%)	388 (9.2%)	398 (9.4%)	170 (4.4%)	344 (6.0%)	
**Men with non-resistant AH**	1,161 (27.3%)	1,138 (25.3%)	1,235 (22.9%)	1,055 (23.1%)	1,202 (23.7%)	1,210 (24.3%)	1,113 (21.5%)	801 (18.9%)	757 (18.0%)	917 (23.6%)	1,470 (25.6%)	
**Men with resistant AH**	159 (3.7%)	208 (4.6%)	185 (3.4%)	132 (2.9%)	145 (2.9%)	89 (1.8%)	122 (2.4%)	61 (1.4%)	54 (1.3%)	51 (1.3%)	128 (2.2%)	

Table S2. General characteristics in men with and without arterial hypertension

	Men without AH (0) (*n*=27,481)	Men with undiagnosed AH (1) (*n*=6,583)	Men with diagnosed not treated AH (2) (*n*=4,555)	Men with non-resistant AH (3) (*n*=12,059)	Men with resistant AH (4) (*n*=1,334)	*p* overall	0 vs. 1	0 vs. 2	0 vs.3	0 vs. 4	1 vs.2	1 vs. 3	1 vs. 4	2 vs. 3	2 vs. 4	3 vs. 4
	Mean (SD)	Mean (SD)	Mean (SD)	Mean (SD)	Mean (SD)	*p*-values										
**Age (y)**	46.2 (4.43)	46.6 (4.42)	47.1 (4.33)	47.7 (4.34)	48.4 (4.19)	<0.001	<0.001	<0.001	<0.001	<0.001	<0.001	<0.001	<0.001	<0.001	<0.001	<0.001
**SBP (mmHg)**	123 (7.24)	139 (8.83)	148 (15.0)	143 (15.7)	151 (17.1)		<0.001	<0.001	<0.001	<0.001	<0.001	<0.001	<0.001	<0.001	<0.001	<0.001
**DBP (mmHg)**	77.7 (5.46)	87.7 (6.96)	92.5 (9.69)	88.2 (10.0)	92.4 (10.4)		<0.001	<0.001	<0.001	<0.001	<0.001	<0.001	<0.001	<0.001	0.994	<0.001
**Heart rate (BPM)**	70.1 (8.25)	73.1 (9.66)	74.9 (10.7)	73.0 (9.29)	73.9 (9.39)		<0.001	<0.001	<0.001	<0.001	<0.001	0.816	0.057	<0.001	<0.001	0.007
**BMI (kg/m²)**	26.4 (3.87)	27.9 (4.31)	29.3 (4.99)	30.3 (5.07)	32.8 (5.57)		<0.001	<0.001	<0.001	<0.001	<0.001	<0.001	<0.001	<0.001	<0.001	<0.001
**Waist (cm)**	92.8 (10.7)	97.1 (11.4)	101 (12.6)	103 (12.9)	109 (13.3)		<0.001	<0.001	<0.001	<0.001	<0.001	<0.001	<0.001	<0.001	<0.001	<0.001
**Glucose (mmol/l)**	5.38 (1.01)	5.60 (1.32)	5.72 (1.55)	5.83 (1.61)	6.12 (1.74)		<0.001	<0.001	<0.001	<0.001	<0.001	<0.001	<0.001	<0.001	<0.001	<0.001
**TC (mmol/l)**	5.69 (1.17)	5.87 (1.16)	6.03 (1.24)	5.93 (1.24)	5.98 (1.22)		<0.001	<0.001	<0.001	<0.001	<0.001	0.008	0.030	<0.001	0.575	0.713
**LDL-C (mmol/l)**	3.62 (1.04)	3.73 (1.05)	3.82 (1.09)	3.77 (1.06)	3.80 (1.05)		<0.001	<0.001	<0.001	<0.001	<0.001	0.081	0.152	0.057	0.981	0.848
**HDL-C (mmol/l)**	1.43 (0.45)	1.42 (0.49)	1.41 (0.46)	1.33 (0.42)	1.26 (0.36)		0.636	0.013	<0.001	<0.001	0.464	<0.001	<0.001	<0.001	<0.001	<0.001
**TG (mmol/l)**	1.55 (1.22)	1.74 (1.40)	1.94 (1.51)	2.04 (1.64)	2.32 (1.82)		<0.001	<0.001	<0.001	<0.001	<0.001	<0.001	<0.001	<0.001	<0.001	<0.001

Abbreviations: AH – arterial hypertension, SBP – systolic blood pressure, DBP – diastolic blood pressure, BPM – beats per minute, BMI – body mass index, TC – total cholesterol, LDL-C – low-density lipoprotein cholesterol, HDL-C – high-density lipoprotein cholesterol, TG – triglycerides.

Table S3. Cardiovascular risk factors in men with and without arterial hypertension.

	Men without AH (0) (*n*=27,481)	Men with undiagnosed AH (1) (*n*=6,583)	Men with diagnosed not treated AH (2) (*n*=4,555)	Men with non-resistant AH (3) (*n*=12,059)	Men with resistant AH (4) (*n*=1,334)	*p* overall	0 vs. 1	0 vs. 2	0 vs.3	0 vs. 4	1 vs.2	1 vs. 3	1 vs. 4	2 vs. 3	2 vs. 4	3 vs. 4
	n (%)	*n* (%)	*n* (%)	*n* (%)	*n* (%) (SD)	*p*-values										
**Smoking history**	10,926 (39.8%)	2,829 (43.0%)	2,041 (44.8%)	4,187 (34.7%)	442 (33.1%)	<0.001	<0.001	<0.001	<0.001	<0.001	0.064	<0.001	<0.001	<0.001	<0.001	0.26
**Unhealthy diet**	15,838 (57.6%)	4,168 (63.3%)	3,234 (71.0%)	8,232 (68.9%)	999 (74.9%)		<0.001	<0.001	<0.001	<0.001	<0.001	<0.001	<0.001	0.001	0.006	<0.001
**Physical inactivity**	10,852 (39.5%)	2,992 (45.5%)	2,335 (51.3%)	6,736 (55.9%)	849 (63.6%)		<0.001	<0.001	<0.001	<0.001	<0.001	<0.001	<0.001	<0.001	<0.001	<0.001
**Diabetes**	1,729 (6.29%)	583 (8.86%)	728 (16.0%)	2,227 (18.5%)	346 (25.9%)		<0.001	<0.001	<0.001	<0.001	<0.001	<0.001	<0.001	<0.001	<0.001	<0.001
**Obesity**	4,360 (15.9%)	1,852 (28.1%)	1,892 (41.5%)	5,632 (46.7%)	891 (66.8%)		<0.001	<0.001	<0.001	<0.001	<0.001	<0.001	<0.001	<0.001	<0.001	<0.001
**Dyslipidemia**	24,096 (87.7%)	6,004 (61.2%)	4,253 (93.4%)	11,348 (94.1%)	1,274 (95.5%)		<0.001	<0.001	<0.001	<0.001	<0.001	<0.001	<0.001	0.084	0.007	0.048
**High TG**	7,825 (28.5%)	2,358 (35.8%)	1,978 (43.4%)	5,755 (47.7%)	758 (56.8%)		<0.001	<0.001	<0.001	<0.001	<0.001	<0.001	<0.001	<0.001	<0.001	<0.001
**Low HDL-C**	8,609 (31.3%)	2,274 (34.5%)	1.,652 (36.3%)	5,046 (41.8%)	628 (47.1%)		<0.001	<0.001	<0.001	<0.001	0.064	<0.001	<0.001	<0.001	<0.001	<0.001
**High LDL-C**	19,957 (72.6%)	5,018 (76.2%)	3,542 (77.8%)	9,319 (77.3%)	1,058 (79.3%)		<0.001	<0.001	<0.001	<0.001	0.104	0.134	0.033	0.521	0.271	0.134
**High TC**	19,779 (72.0%)	5,137 (78.0%)	3,646 (80.0%)	9,450 (78.4%)	1,064 (79.8%)		<0.001	<0.001	<0.001	<0.001	0.023	0.683	0.249	0.032	0.85	0.317

Abbreviations: TG – triglycerides, HDL-C – high-density lipoprotein cholesterol, LDL-C – low-density lipoprotein cholesterol, TC – total cholesterol.
